# Effect of cardio-metabolic risk factors on all-cause mortality among HIV patients on antiretroviral therapy in Malawi: A prospective cohort study

**DOI:** 10.1371/journal.pone.0210629

**Published:** 2019-01-17

**Authors:** Alemayehu Amberbir, Victor Banda, Victor Singano, Alfred Matengeni, Colin Pfaff, Zahra Ismail, Theresa J. Allain, Adrienne K. Chan, Sumeet K. Sodhi, Joep J. van Oosterhout

**Affiliations:** 1 Dignitas International, Zomba, Malawi; 2 Pirimiti Rural Hospital, Pirimiti, Malawi; 3 Department of Medicine, College of Medicine, University of Malawi, Blantyre, Malawi; 4 Division of Infectious Diseases, Department of Medicine, Sunnybrook Health Sciences Centre, University of Toronto, Toronto, Canada; 5 Department of Family and Community Medicine, Toronto Western Hospital, University Health Network, Toronto, Canada; Boston University, UNITED STATES

## Abstract

**Background:**

Cardiovascular disease (CVD) risk among people living with HIV is elevated due to persistent inflammation, hypertension and diabetes comorbidity, lifestyle factors and exposure to antiretroviral therapy (ART). Data from Africa on how CVD risk affects morbidity and mortality among ART patients are lacking. We explored the effect of CVD risk factors and the Framingham Risk Score (FRS) on medium-term ART outcomes.

**Methods:**

A prospective cohort study of standardized ART outcomes (Dead, Alive on ART, stopped ART, Defaulted and Transferred out) was conducted from July 2014—December 2016 among patients on ART at a rural and an urban HIV clinic in Zomba district, Malawi. The primary outcome was Dead. Active defaulter tracing was not done and patients who transferred out and defaulted were excluded from the analysis. At enrolment, hypertension, diabetes and dyslipidemia were diagnosed, lifestyle data collected and the FRS was determined. Cox-regression analysis was used to determine independent risk factors for the outcome Dead.

**Results:**

Of 933 patients enrolled, median age was 42 years (IQR: 35–50), 72% were female, 24% had hypertension, 4% had diabetes and 15.8% had elevated total cholesterol. The median follow up time was 2.4 years. Twenty (2.1%) patients died, 50 (5.4%) defaulted, 63 (6.8%) transferred out and 800 (85.7%) were alive on ART care (81.7% urban vs. 89.9% rural). In multivariable survival analysis, male gender (aHR = 3.28; 95%CI: 1.33–8.07, p = 0.01) and total/HDL cholesterol ratio (aHR = 5.77, 95%CI: 1.21–27.32; p = 0.03) were significantly associated with mortality. There was no significant association between mortality and hypertension, body mass index, central obesity, diabetes, FRS, physical inactivity, smoking at enrolment, ART regimen and WHO disease stage.

**Conclusions:**

Medium-term all-cause mortality among ART patients was associated with male gender and elevated total/HDL cholesterol ratio.

## Introduction

The burden of non-communicable diseases (NCDs) is increasing in Africa.[1] The prevalence of hypertension and diabetes in the general population of Malawi was estimated to be 33% and 6% respectively and both conditions were mostly undiagnosed and therefore untreated.[2] NCDs are also common among patients in HIV care.[3] In 2014, we performed a survey in an urban and a rural HIV clinic showing that 24% of adult Malawians enrolled in HIV care had hypertension and 4% had diabetes.[4] Because the life expectancy of persons living with HIV (PLHIV) has improved dramatically due to increasing antiretroviral therapy (ART) coverage, [5] the impact of cardiovascular diseases (CVD) on their morbidity and mortality has increased.[6]

Cardiovascular risk among PLHIV may be increased due to persistent inflammation, exposure to antiretroviral drugs (ARVs), traditional CVD risk factors and other reasons.[6] Of multiple available CVD risk scores, the most used is the Framingham risk score [FRS].[7] Solid validation of the FRS and other CVD risk scores with clinical endpoints is lacking for African populations of PLHIV and to our knowledge there are no prospective studies about the correlation of CVD risk factors with clinical outcomes in African adults on ART.

We have, therefore, explored the effects of cardiovascular risk factors on medium-term mortality in a prospective cohort study in Zomba district, Malawi.

## Methods

### Study setting

Between July and October 2014 we consecutively enrolled HIV infected adults (aged 18 years or older) at a rural and an urban HIV clinic in Zomba district, Malawi. The clinics are located in Zomba district, southern Malawi, where HIV prevalence is around 14.5% in the 15–49 year age group.[5] We obtained information about the HIV treatment history and assessed life style factors relevant for CVD such as smoking status and physical activity with STEPS methodology.[8]

### Data collection and clinical definitions

At enrolment, hypertension, diabetes and dyslipidemia were diagnosed, general and abdominal obesity were measured and the FRS was determined. Details of the methods have been described in detail elsewhere.[4] We diagnosed hypertension if a systolic blood pressure (SBP) of ≥140 mm Hg and/or diastolic blood pressure (DBP) ≥90 mmHg was confirmed at two follow-up visits or if the patient was on antihypertensive medication. Hypertension was categorized as stage I (SBP 140–159 and/or DBP 90–99 mmHg), stage II (SBP 160–179 and/or DBP 100–109 mmHg) and stage III (SBP ≥180 and/or DBP ≥110 mmHg). Diabetes was defined as a fasting blood glucose level >126mg/dl or random blood glucose level >200 mg/dl and confirmed at a second visit, or if a patient was on medication for diabetes. The FRS is valid for patients aged 20 years and older. FRS was calculated as previously described and incorporates sex, age, current smoking status, total cholesterol, HDL-cholesterol, treatment for hypertension, and systolic blood pressure.[9] FRS provides a percentage expressing the chance of a major cardiovascular event or death in the next 10 years. Body mass index (BMI) was categorized as underweight (<18.5 Kg/m2), normal (18.5 to 24.9 Kg/m2), overweight (25.0 to 29.9 Kg/m2) or obese (>30.0 Kg/m2) according to the WHO thresholds.[10] Central obesity was defined as waist-to-hip ratio (waist circumference/hip circumference) of ≥0.95 for men and ≥0.85 for women.[10]

The 933 patients who were enrolled in 2014 [4] were then followed for two and half years during routine ART service delivery. Standard ART outcomes were extracted from the electronic monitoring systems at each site at the end of December 2016: Dead, Alive on ART, Stopped ART, Defaulted (defined as not seen in the clinic for more than two months after the patient has known to have run out of medication and not known to have died, transferred or stopped medication), and Transferred out (defined as known to have transferred to another ART clinic). The national HIV programme monitoring system in Malawi has been described in depth elsewhere.[5]

### Data management and statistical analysis

ART data were checked for consistency against routinely used individual ART master cards and ART registers.[11] All data were then entered in a single Access database, cleaned, anonymized, coded and merged for analysis using Stata 13 (Statacorp, College Station Texas, USA). The primary study outcome was Dead. Patients with standard ART outcomes Transferred out and Defaulted were excluded from the analysis as we were unable to ascertain their outcome and active defaulter tracing was not done. The 10-year FRS risk of death and/or major CVD event was categorized as 0–5% or >5%. ART outcomes were first tabulated by health facility setting (rural vs. urban). Cardio-metabolic variables and FRS as determined at enrolment were then described by the primary study outcome Dead. The effects of cardio-metabolic variables on outcome Dead were explored using survival analysis. Crude hazard ratios with 95% confidence intervals were computed using Cox-proportional hazards analysis to determine univariate associations between cardio-metabolic variables and standard ART outcomes. In a multivariate analysis, we controlled for the potential confounders, reporting adjusted hazard with 95% confidence interval. Age is universally acknowledged as a crucial CVD risk factor. Urbanization is a recognized driver of CVD, the prevalence of cardiovascular risk factors is believed to be different in rural and urban settings in Africa [12] and some unmeasured life style aspects that are relevant for CVD risk may differ between rural and urban dwellers. Urban and rural study site and age were therefore included in the model as *a priori* confounders. The final model included study site, age, sex, diagnosis of hypertension and total HDL/total cholesterol ratio. Variables with missing data were fitted in the model with a missing category. Overall p-values and p-value for trend were reported as appropriate using the likelihood ratio test (LHR).

### Ethics approval and consent to participate

The study was approved by College of Medicine Research and Ethics Committee, Blantyre, Malawi (approval number P.07/16/1985). This included permission to extract routinely collected ART outcome data from electronic monitoring systems. The study adhered to the principles of the Declaration of Helsinki. All enrolled participants gave their informed written consent.

## Results

### Description of the study cohort

**Fig 1:**
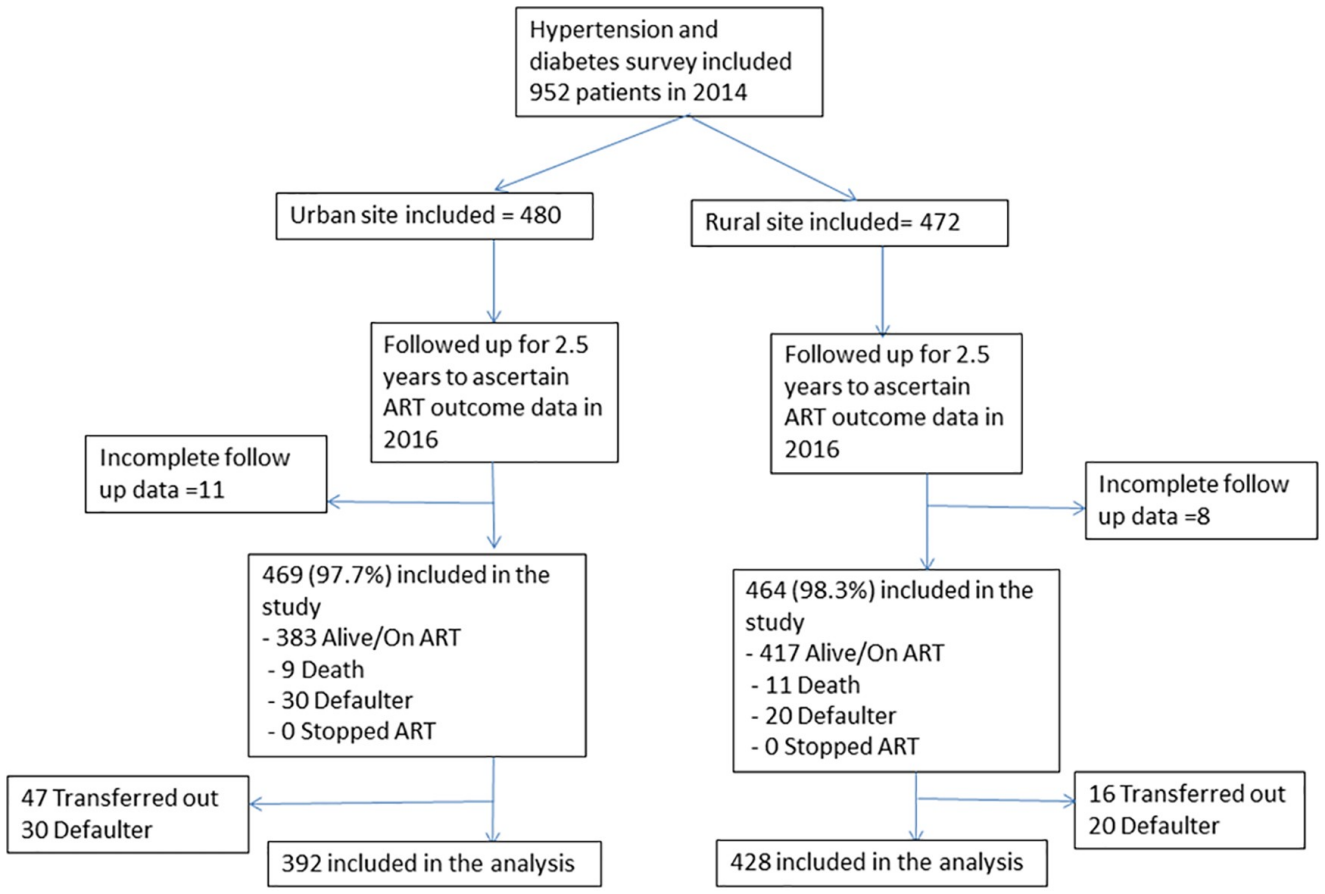
Study flow chart of patients enrolled at two HIV clinics in Zomba district, Malawi.

Of 952 (urban 480, rural 472) patients enrolled at baseline, 19 had incomplete follow up data at two and half years and therefore 933 (98%) were included in the study, as shown in Fig 1. Of these patients, 63 Transferred out to another facility and 50 defaulted, leaving 820 patients for inclusion in the analysis. After two and half years of follow up, 800 (85.7%) were Alive on ART. Twenty had the outcome Dead during follow up, resulting in a mortality rate of 2.4% (95%CI: 1.6% to 3.6%). There was a significant difference in standardized ART outcomes between the study clinics (Table 1), mainly determined by a larger number of Transfer out outcomes in the urban clinic.

**Table 1:** Description of ART outcome by HIV clinic, Zomba district, Malawi.

ART outcome (N = 933)	Total n (%)	Rural (N = 464) n (%)	Urban (N = 469) n (%)	P-value
Dead	20 (2.1)	11 (2.4)	9 (1.9)	<0.01
Defaulted	50 (5.4)	20 (4.3)	30 (6.4)	
Alive on ART	800 (85.7)	417 (89.9)	383 (81.7)	
Transferred out	63 (6.8)	16 (3.5)	47 (10.0)	
Stop ART	0 (0)	0 (0)	0 (0)	

### Cardio-metabolic factors at enrolment

A majority of participants were female 589 (71.8%). The median age was 42 years (IQR 35–50). The majority, 685 (85.4%) were on a single tablet formulation of tenofovir, lamivudine and efavirenz. The median ART duration was 48 months (IQR 24–71), the median CD_4_ count at ART initiation was 223 cells/mm^3^ (IQR 130–283), and 512 (63.8%) were in WHO disease stage I/II at ART initiation. Vigorous physical activity was reported by 743 (90.6%), 28 (3.4%) were current smokers and 143 (17.4%) were overweight or obese. Hypertension was diagnosed in 195 (23.8%), diabetes in 35 (4.3%), elevated total cholesterol in 82 (17.1%), reduced level of HDL cholesterol in 73 (15.2%), elevated total/HDL cholesterol in 18 (3.7%), elevated triglycerides in 139 (28.9%) and 46 (9.6%) had a 10-year FRS risk >5% (Table 2).

**Table 2:** Cardiovascular risk factors by outcome Dead among ART patients in Malawi.

Risk factors	Total n/N (%)	Dead n (%)	Crude Hazard ratio (95% CI)	P-value
*Study site (N = 820)*				
Rural	428 (52.2)	11 (2.6)	1	0.37
Urban	392 (47.8)	9 (2.3)	0.90 (0.37, 2.15)	
*Sex (N = 820)*				
Female	589 (71.8)	9 (1.5)	**1**	**0.01**
Male	231 (28.2)	11 (4.8)	**3.16 (1.31, 7.64)**	
*Age in years (N = 820)*				0.96† / 0.96*
≤30	73 (8.9)	2 (2.7)	1	
31–45	435 (53.1)	10 (2.3)	0.82 (0.18, 3.76)	
>45	312 (38.1)	8 (2.6)	0.92 (0.20, 4.33)	
*Diagnosis of hypertension (N = 820)*				0.51
No	625 (76.2)	14 (2.2)	1	
Yes	195 (23.8)	6 (3.1)	1.37 (0.53, 3.58)	
*Diagnosis of diabetes (N = 820)*				-
No	785 (95.7)	20 (2.6)	-	
Yes	35 (4.3)	0 (0.0)	-	
*Total cholesterol (N = 481)*				0.10
Normal	399 (83.0)	7 (1.8)	1	
Elevated	82 (17.1)	4 (4.9)	2.82 (0.82, 9.62)	
*HDL cholesterol (N = 481)*				0.06
Normal	408 (84.8)	7 (1.7)	1	
Decreased	73 (15.2)	4 (5.5)	3.24 (0.95, 11.05)	
*Total/HDL cholesterol (N = 481)*				**0.02**
Normal	463 (96.3)	9 (1.9)	1	
Elevated	18 (3.7)	2 (11.1)	**5.97 (1.29, 27.62)**	
*Triglycerides (N = 481)*				0.90
Normal	342 (71.1)	8 (2.3)	1	
Elevated	139 (28.9)	3 (2.2)	0.92 (0.24, 3.46)	
*Framingham risk score >5% (N = 477)*				
No	431 (90.4)	10 (2.3)	1	0.95
Yes	46 (9.6)	1 (2.2)	0.94 (0.12, 7.35)	
*WHO stage at ART initiation (N = 802)*				
Stage I & II	512 (63.8)	14 (2.7)	1	0.37
Stage III & IV	290 (36.2)	5 (1.7)	0.63 (0.23, 1.74)	
*ART regimen (N = 802)*				0.86
5A	685 (85.4)	16 (2.3)	1	
2A	93 (11.6)	2 (2.2)	0.92 (0.21, 4.00)	
Other	24 (3.0)	1 (2.4)	1.80 (0.24, 13.55)	
*ART duration in months (N = 802)*				
<24	198 (24.7)	4 (2.0)	1	0.71
≥24	604 (75.3)	15 (2.5)	1.23 (0.41, 3.71)	
*Body mass index (Kg/m*^*2*^*) (N = 820)*				0.16† / 0.35*
Normal	584 (71.2)	15 (2.6)	1	
Underweight	93 (11.3)	4 (4.3)	1.67 (0.55, 5.02)	
Overweight/obesity	143 (17.4)	1 (0.7)	0.27 (0.04, 2.05)	
*Waist—to-hip ratio (N = 796)*				
Normal	580 (72.9)	13 (2.2)	1	0.66
High	216 (27.1)	6 (2.8)	1.24 (0.47, 3.27)	
*Physical activity (N = 820)*				0.38
Moderately active	77 (9.4)	3 (3.9)	1	
Vigorously active	743 (90.6)	17 (2.3)	0.58 (0.17, 1.99)	
*Smokers at baseline (N = 820)*				-
No	792 (96.6)	20 (2.5)	-	
Yes	28 (3.4)	0 (0)	-	

2A, generic single tablet formulation of zidovudine, lamivudine, nevirapine; 5A, generic single tablet formulation of tenofovir, lamivudine, efavirenz (regimens coded as in national HIV management guidelines)

^†^Overall p value (Likelihood ratio test);

*P value for trend

### Association of cardio-metabolic factors with mortality; results of survival analysis and Cox regression modeling

In univariate analysis the outcome Dead was associated with male gender and elevated total/HDL cholesterol, but no significant associations were found with rural/urban study site, age, hypertension, diabetes, FRS, ART regimen, duration on ART, WHO disease stage, body mass index, central obesity, current smoking or and physical activity level (Table 2).

In a multivariable Cox regression analysis, male gender (aHR = 3.28; 95%CI: 1.33–8.07, p = 0.01) and elevated total/HDL cholesterol ratio (aHR = 5.77, 95%CI: 1.21–27.32; p = 0.03) were significantly associated with outcome Dead (Table 3).

**Table 3:** Cox-proportional hazards analysis: Independent association of lifestyle and cardiovascular risk factors with outcome Dead among ART patients in Malawi.

*Risk factors*	*Dead n (%)*	*Crude Hazard ratio (95% CI)*	Adjusted Hazard ratio (95% CI)**	*P-value*
*Study site (N = 820)*				
Rural	11 (2.6)	1	1	0.86
Urban	9 (2.3)	0.90 (0.37, 2.15)	0.92 (0.38, 2.24)	
*Sex (N = 820)*				
Female	9 (1.5)	**1**	**1**	**0.01**
Male	11 (4.8)	**3.16 (1.31, 7.64)**	**3.28 (1.33, 8.07)**	
*Age in years (N = 820)*				0.79† / 0.58*
≤30	2 (2.7)	1	1	
31–45	10 (2.3)	0.82 (0.18, 3.76)	0.60 (0.13, 2.82)	
>45	8 (2.6)	0.92 (0.20, 4.33)	0.54 (0.11, 2.70)	
*Diagnosis of hypertension (N = 820)*				
No	14 (2.2)	1	1	0.56
Yes	6 (3.1)	1.37 (0.53, 3.58)	1.35 (0.49, 3.68)	
*Total/HDL cholesterol (N = 481)*				**0.03**
Normal	9 (1.9)	**1**	**1**	
Elevated	2 (11.1)	**5.97 (1.29, 27.62)**	**5.77 (1.23, 27.32)**	

^†^Overall p value (Likelihood ratio test)

* P value for trend

** Final model included: study site, sex, age, diagnosis of hypertension and total/HDL-c. Urban and rural study site and age were included in the model as *a priori* confounder

Total/HDL cholesterol variable was fitted in the model with a separate missing category

## Discussion

Cardiovascular disease risk among patients on ART is reported to be increased due to a number of factors including chronic inflammation, immune activation, traditional risk factors and exposure to antiretroviral drugs.[6] In this exploratory study, medium-term all-cause mortality during ART follow up was associated with elevated total/HDL cholesterol ratio and male gender. However, no association was observed with other classic cardio-metabolic and lifestyle risk factors, nor with the FRS.

The roll out of ART in Africa has resulted in a strong reduction in all-cause mortality.[3] Experience from HIV programs has shown that effective reduction in HIV/AIDS related mortality can be achieved through decentralized ART care along with a strengthened health monitoring and evaluation system.[5] Despite these efforts, mortality has remained considerable. In a cohort study in Tanzania mortality was 12% among ART patients and was associated with WHO stage III and IV, lower CD4 counts, severe anemia and male gender. [13] Other recent studies have shown that age (>35 years old), low weight, WHO disease stage IV at ART initiation and poor adherence were predictors of mortality.[14] In our study, all-cause mortality was not associated with the type of facility (urban/rural), age, current ART regimen and duration on ART or WHO disease stage at ART initiation. Observed differences in risk factors for mortality may relate to the study periods. Recent studies after the wider roll out of ART to rural primary health facilities and with a high percentage of patients with early HIV infection are likely to have different mortality and risk factor patterns compared to older studies [13].

We found a significant association between male gender and risk of mortality. In the Malawi HIV programme, there is a female preponderance among patients on ART similar to other countries in the region [15]. The percentage of women in our study (72%) was somewhat higher than in the Malawi ART population and the populations in care at the two study clinics (around 64%), possibly because men less often visit clinics and may be less willing to participate in research studies. This may have caused a lower overall mortality in the study population but the observed increased mortality risk of men is unlikely to be biased given that it has been reported consistently in African studies.[14, 16,17] Various explanations, including delay in HIV diagnosis, late reporting for care and hence delay in initiating ART, and poor adherence to treatment, have been suggested.[17]

Our study showed a six-fold increased risk of mortality among those with an elevated total/HDL cholesterol ratio compared with normal lipid profiles within the follow up period. Measured at the baseline, dyslipidemia was moderately common in our cohort: elevated TC (15.5%), reduced HDL-c (15.9%), elevated TG (28.7%) and elevated TC/HDL-c ratio (3.8%) [18]. Few studies have compared the CVD risk of Malawian adults on ART compared to the general population. A cross sectional study showed higher prevalence of diabetes and low-density lipoprotein cholesterol in Malawians on long-term ART than in HIV negative controls, especially in the higher age groups. The prevalence of hypertension was similarly high in patients on ART and HIV negatives [19]. This study underlines the potential strong impact of CVD on mortality of Malawians on ART. A meta-analysis of published articles from sub-Saharan Africa reported that HIV infection was associated with hypertriglyceridaemia and lower HDL levels whereas ART use was associated with higher HDL and LDL levels.[20] A Ugandan study among ART patients reported a high burden of (58%) metabolic syndrome (defined in the study as at least two of the following: fasting blood sugar >100mg/dl, blood pressure of ≥ 130/85mm Hg, triglycerides ≥150mg/dl, HDL ≤ 40mg/dl, or waist circumference ≥ 94cm in males or ≥ 80cm in females).[21] A study from a South African population on ART also reported unfavorable lipid profile changes and increased CVD risk, with older age, obesity, lower CD4 count and non-tenofovir containing regimens being risk factors for elevated cholesterol.[22] Despite the increasing notion that CVD risk is raised in PLHIV, African studies looking at determinants of mortality among ART patients have not included cardiovascular risk factors.

Although current guidelines in Africa do not recommend regular screening for lipids in ART clinics, it may be beneficial to determine lipid profiles in patients on ART to enable comprehensive CVD risk assessment, especially given the high prevalence of the metabolic syndrome. On the other hand, scarce resources limit the availability of treatment of dyslipidemia and the high burden of care at busy HIV clinics in sub-Saharan Africa would make additional screening and integrated treatment challenging. The feasibility and cost-effectiveness of adding regular screening of lipids and integrated treatment of dyslipidemia at African ART clinics requires further study.

Cardiovascular disease contributes to the morbidity and mortality of HIV patients [6] although few studies from the region have reported CVD outcomes in HIV populations. A recent study showed that the increasing burden of ischemic stroke can be attributed to HIV infection and the early phase of ART, especially in younger patients with stroke.[23] In this study, most classic CVD risk factors including the FRS were not associated with all-cause mortality. Despite the importance of overall cardiometabolic risk in ART patients, little is known about the consequences of CVD risk scores on mortality or clinical events in HIV populations. The FRS was derived in the Unites States and is traditionally used in western cohorts but it has not been validated in African patients.[7] There is a pressing need to determine the applicability of existing CVD risk scores in African ART patients through large and well powered prospective studies.

The strengths of our cohort study include the use of validated WHO STEPS methods [8] to provide comprehensive cardiometabolic risk profiles measured at the baseline and reported elsewhere [4, 18]. We used various HIV and ART related clinical information in our analysis and our final model was adjusted for relevant potential confounders. The study also had a low rate of loss to follow up. To our knowledge this is the first study that prospectively explored the effects of cardiovascular risk factors on mortality among patients on ART in sub-Saharan Africa. It provides initial insight in to the role of monitoring CVD risk factors in HIV populations in resource poor settings.

The findings of this study should be interpreted in the light of its limitations. We investigated all-cause mortality and could not establish the cause of death; therefore deaths may have had non-cardiovascular causes. The duration of follow up and the amount of person-years of observation was fairly small for a study that investigates risk factors of mortality. As a result, we may not have found associations between cardiometabolic risk factors and mortality due to lack of statistical power. In addition, we were unable to diagnose non-fatal cardiovascular events as we used routinely collected ART outcome information from national HIV programme monitoring tools that do not include reliable morbidity data. We measured CVD risk factors at enrolment only and some traditional risk factors, particularly those related to lifestyle may vary over time. Because of restrictions in funding, serum lipids could only be determined in the first 497 patients. Lipid and FRS data were therefore not available from all patients, although bias associated with this testing was likely to be non-differential. Defaulted patients were excluded from the analysis as we could not assess their vital status and active defaulter tracing was not done. In an earlier stage of the Malawi HIV programme, when patients started ART at more advanced HIV disease, around a third of defaulters had died.[4] Although the percentage of deaths among defaulters at the time of our study is likely to be much lower, missing deaths among those with the outcome Defaulted may have introduced a degree of bias.

## Conclusions

In this study from two large Malawian HIV clinics, medium-term all-cause mortality among ART patients was associated with male gender and elevated total/HDL cholesterol ratio, but not with the FRS and most classic CVD risk factors. Larger studies with longer follow up that investigate CVD risk and clinical CVD outcomes in African ART populations are urgently needed. Efforts to combine HIV care along with the provision of CVD risk factor screening and management are underway but require a stronger evidence base.
